# Optimal lipid-lowering therapy in patients who were functionally deferred percutaneous coronary intervention

**DOI:** 10.1038/s41598-023-37988-z

**Published:** 2023-07-05

**Authors:** Takuro Abe, Kensuke Shimazaki, Tetsu Moriyama, Akira Murohashi, Yuji Iwanami, Akihito Sasaki, Katsumi Saito, Kentaro Jujo

**Affiliations:** 1 Department of Cardiology, Nishiarai Heart Center Hospital, Tokyo, Japan; 2 Department of Cardiovascular Surgery, Nishiarai Heart Center Hospital, Tokyo, Japan; 3 Department of Cardiology, Saitama Medical University, Saitama Medical Center, 1981 Kamoda, Kawagoe-shi, Saitama, 350-8550 Japan

**Keywords:** Cardiovascular biology, Interventional cardiology, Risk factors

## Abstract

Deferral of percutaneous coronary intervention (PCI) for functionally insignificant stenosis, defined as fractional flow reserve (FFR) > 0.80, is associated with favorable long-term prognoses. The lower-the-better strategy for low-density lipoprotein cholesterol (LDL-C) management is an established non-angioplasty therapy to improve the clinical outcomes of patients undergoing PCI. We examined the optimal LDL-C management in cases of intermediate coronary stenosis with deferred PCI on the basis of FFR values. This observational study included 273 consecutive patients with a single target vessel and deferred PCI with an FFR > 0.80. Patients with an FFR of 0.81–0.85 (n = 93) and those with FFR > 0.85 (n = 180) were classified into the lower (< 100 mg/dL) and higher (≥ 100 mg/dL) LDL-C groups. The endpoint was major adverse cardiovascular and cerebrovascular events (MACCE), including death, non-fatal myocardial infarction, ischemic stroke, heart failure hospitalization, and unplanned revascularization. Patients with an FFR of 0.81–0.85 had a significantly higher MACCE rate than those with an FFR > 0.85 (log-rank, *p* = 0.003). In patients with an FFR of 0.81–0.85, the lower LDL-C group showed a significantly lower MACCE rate than the higher LDL-C group (log-rank, *p* = 0.006). However, the event rate did not differ significantly between the two groups in patients with FFR > 0.85 (log-rank, *p* = 0.84). Uncontrolled LDL-C levels were associated with higher MACCE rates in cases with deferred PCI due to an FFR of 0.81–0.85. This high-risk population for adverse cardiovascular events should receive strict LDL-C-lowering therapy.

## Introduction

The fractional flow reserve (FFR) is an established diagnostic parameter for identifying myocardial ischemia with coronary artery stenosis^1^. The extent of myocardial ischemia measured using a coronary pressure wire is an important determinant of outcomes in patients with stable coronary artery disease (CAD). FFR has been previously shown to safely indicate whether coronary stenosis requires revascularization by percutaneous coronary intervention (PCI) plus medical therapy or medical therapy alone^2–5^. Current guidelines recommend FFR measurement before revascularization in patients with chronic coronary syndrome without evidence of ischemia from non-invasive testing^6^.

A number of large clinical trials and meta-analyses have established the “the lower, the better” strategy for low-density lipoprotein cholesterol (LDL-C) management in patients after PCI^7–9^. However, the clinical influence of lipid-lowering therapy after deferral of PCI in patients with stenotic lesions has not been well evaluated. Therefore, in clinical practice, patients with angiographically intermediate coronary artery stenosis often receive insufficient lipid-lowering therapy. We aimed to stratify the risk of adverse clinical events in cases where PCI was deferred on the basis of FFR values, focusing on the LDL-C levels during the follow-up period.

## Methods

### Study design, population, and endpoints

This observational study initially included consecutive patients with intermediate coronary artery stenosis who underwent FFR testing at Nishiarai Heart Center Hospital between June 2012 and December 2019. Indications for coronary angiography in our study were suspicion of chronic coronary syndrome, encompassing stable effort angina and silent myocardial ischemia. We excluded acute coronary syndrome (ACS) cases as non-culprit lesions in ACS are deemed vulnerable, making physiological assessments less applicable for intervention guidance. Patients suspected of stable effort angina showed symptoms such as angina or exertional dyspnea, while silent myocardial ischemia was inferred from abnormalities on electrocardiogram suggestive of ischemia, myocardial wall motion anomalies, stress test positivity, or severe coronary artery calcification. In cases with visually estimated 50–90% intermediate coronary stenosis and absent objective ischemia evidence (e.g., stress myocardial scintigraphy or exercise stress test)^6^, FFR measurements were undertaken upon the consensus of at least two expert cardiologists. After exclusion of patients with coronary stenosis and FFR ≤ 0.80, those with multiple stenoses, those without follow-up observations, and those receiving regular hemodialysis, patients with a single target vessel whose PCI had been deferred due to FFR values > 0.80 were ultimately enrolled in this study. The enrolled patients were divided into two groups depending on the cut-off FFR value of 0.85, as referred to in a previous report^5^. Patients in each of these FFR-based groups were further classified into two groups based on the LDL-C level at 1 year after the FFR measurement–the lower LDL-C group (< 100 mg/dL) and the higher LDL-C group (≥ 100 mg/dL)–on the basis of the target LDL-C value for patients who needed secondary prevention of adverse cardiovascular events under the guidelines^10,11^.

The endpoint of the current study was a major adverse cardiovascular and cerebrovascular event (MACCE), including death, non-fatal myocardial infarction, non-fatal stroke, hospitalization due to worsening of heart failure, and unplanned revascularization after the FFR measurement referred to the previous trials about lipid lowering therapy and FFR^12^. This study included only unplanned revascularization events that targeted the vessel that had been assessed with FFR measurements in the endpoint analysis. This study complied with the principles of the Declaration of Helsinki. Seiwakai Medical Ethics Committee approved this study protocol and waived the written informed consent because this study was a retrospective observational design.

### Coronary angiography and FFR measurement

Coronary angiography was performed using a guiding catheter via the trans-radial or trans-femoral artery approach with an Allura Xper FD10 (PHILIPS, Amsterdam, the Netherlands) radioscopy system. Coronary pressure measurement was performed using a 0.014-inch sensor-tipped guidewire (Pressure Wire: St. Jude Medical, Minnesota, United States; OptoWire: ZEON MEDICAL INC, Tokyo, Japan) introduced through a 5–7-Fr guiding catheter. The pressure wire was calibrated and advanced into the coronary artery and positioned distal to the stenosis as far as possible. Hyperemia was induced by continuous intravenous administration of 150 µg/kg/min of adenosine. FFR was calculated as the ratio of the mean distal coronary pressure measured by the pressure wire to the mean aortic pressure measured by the guiding catheter during maximal hyperemia.

### Data collection and follow-up

For each patient, we collected data pertaining to the following parameters at the time of FFR measurement: (1) vital signs, (2) medical history, (3) transthoracic echocardiography, (4) quantitative coronary angiography (QCA), and (5) laboratory tests, including hemoglobin, albumin, renal function (creatinine and estimated glomerular filtration rate [eGFR]), B-type natriuretic peptide (BNP), glycated hemoglobin (HbA1c), total cholesterol (T-C), LDL-C, high-density lipoprotein cholesterol (HDL-C), and triglyceride (TG). Oral medications and laboratory values at the time of FFR measurement and one year after the session were compared among the subgroups. QCA was performed using the Cardiovascular Angiography Analysis System (Pie Medical Imaging). Information about adverse clinical events, including death, non-fatal myocardial infarction, ischemic stroke, hospitalization due to worsening of heart failure, unplanned revascularization, and date of events, was collected by the cardiologist from medical records. The endpoints were analyzed on a per-patient basis.

### Statistical analysis

The values are expressed as the mean and standard deviation, median and interquartile, and percentiles. The independent Student’s t-test and the non-parametric equivalent Mann–Whitney U test were used to compare the groups in relation to the continuous variables, and the chi-squared test and Fischer’s exact test were used to compare the groups with respect to the categorical variables, as appropriate. MACCE was evaluated using Kaplan–Meier analysis, and differences between groups were assessed using the log-rank test. Univariate and multivariable analyses were performed using the Cox proportional hazards regression model to evaluate the predictors of MACCE. Variables with a *p*-value < 0.1 in the univariate analyses were included in the subsequent multivariable analysis. We also checked for collinearity among the variables included in the model using variance inflation factor (VIF) analysis, and variables without significant collinearity (VIF < 2) were included in our multivariable model. With respect to the primary endpoint, potential interactions between the FFR (FFR 0.81–0.85 or FFR > 0.85) and LDL-C groups (lower or higher LDL-C groups) were evaluated using logistic regression analysis. Statistical significance was set at a two-sided *p*-value of < 0.05. Statistical analyses were performed using R software, version 3.3.0 (R Foundation for Statistical Computing, Vienna, Austria).

## Results

**Figure 1:**
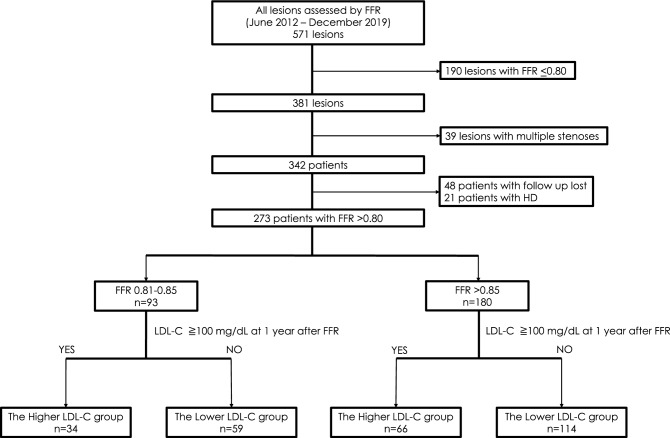
Flow chart of participant recruitment and categorization. FFR, fractional flow reserve; HD, hemodialysis; LDL-C, low-density lipoprotein cholesterol.

The current study initially included 571 consecutive lesions that were assessed using the FFR. We excluded 190 cases with FFR ≤ 0.80, 39 cases that required FFR measurements for multiple stenoses, 48 cases with missing LDL-C data at 1 year after the FFR measurement, and 21 cases involving regular hemodialysis. Thus, we finally analyzed 273 lesions in patients with a single target lesion and FFR > 0.80, including 93 patients with an FFR of 0.81–0.85 and 180 patients with FFR > 0.85 (Fig. 1).

Among the 93 patients with an FFR of 0.81–0.85, 59 and 34 were categorized in the lower and higher LDL-C groups, respectively. Among the patients with FFR > 0.85, 114 and 66 were categorized in the lower and higher LDL-C groups, respectively (Fig. 1).

### Baseline characteristics

#### Entire population

Table 1 presents the baseline characteristics of the study participants. Overall, the median age was 72 years, and 69% were men. Compared with patients with FFR > 0.85, those with an FFR of 0.81–0.85 had a significantly lower prevalence of hypertension and were subjected to FFR tests significantly more frequently in the left main coronary artery or left anterior descending coronary artery. As for QCA data, there was no significant difference between patients with an FFR of 0.81–0.85 and those with FFR > 0.85. Patients with an FFR of 0.81–0.85 were likely to have lower HDL-C and higher TG values at the time of FFR measurement; however, the two FFR populations showed no significant difference in the lipid profile at 1 year after FFR measurement. In both FFR populations, prescription rates of statins increased from baseline to 1 year after the FFR measurement (FFR, 0.81–0.85: 54% to 61%; FFR > 0.85: 45% to 62%), and prescription rates were comparable at all time points in both groups. The statin doses were comparable from FFR up to 1 year after FFR measurement in each group, and the prescription rates of ezetimibe and eicosapentaenoic acid were not significantly different between the FFR measurement and at 1 year after treatment in both groups. None of the patients were using proprotein convertase subtilisin/kexin 9 inhibitors (PCSK9i) at FFR measurement, and only one patient required PCSK9i at 1 year after FFR measurement.

**Table 1:** Patient characteristics.

Variables	FFR 0.81–0.85 n = 93				FFR > 0.85 n = 180				FFR 0.81–0.85 vs. FFR > 0.85
	All	Lower LDL-C	Higher LDL-C	P value	All	Lower LDL-C	Higher LDL-C	P value	P value
		n = 59	n = 34			n = 114	n = 66		
Age, years	70.6 ± 10.3	71.6 ± 10.1	68.9 ± 10.3	0.16	70.2 ± 10.2	71.0 ± 9.4	68.7 ± 11.3	0.28	0.76
Male	64 (69%)	46 (78%)	18 (53%)	0.02	127 (71%)	83 (73%)	44 (67%)	0.40	0.78
BMI, kg/m^2^	24.2 ± 3.2	23.7 ± 3.2	25.1 ± 3.1	0.048	24.3 ± 3.6	24.4 ± 3.3	24.3 ± 4.2	0.87	0.66
Systolic BP, mmHg	153.1 ± 28.9	147.5 ± 25.2	162.8 ± 32.1	0.03	153.6 ± 28.3	154.2 ± 28	152.6 ± 28.7	0.45	0.64
Diastolic BP, mmHg	72.3 ± 14.1	70.6 ± 12.6	75.2 ± 16	0.21	72 ± 14	71.8 ± 12.2	72.2 ± 16.6	0.71	0.92
Heart rate, bpm	68.9 ± 14.3	69.0 ± 16.3	68.7 ± 10.0	0.69	69.8 ± 14.2	69.4 ± 14.2	70.5 ± 14.2	0.37	0.86
Hypertension	64 (69%)	41 (69%)	23 (68%)	> 0.99	147 (82%)	93 (82%)	54 (82%)	> 0.99	0.01
Diabetes	20 (22%)	12 (20%)	8 (24%)	0.59	48 (27%)	29 (25%)	19 (29%)	0.86	0.46
Dyslipidemia	50 (54%)	31 (53%)	19 (56%)	0.83	104 (58%)	70 (61%)	34 (52%)	0.2	0.37
Smoking	27 (29%)	19 (32%)	8 (24%)	0.48	68 (38%)	45 (39%)	23 (35%)	0.52	0.11
Atrial fibrillation	5 (5%)	3 (5%)	2 (6%)	> 0.99	15 (8%)	11 (10%)	4 (6%)	0.42	0.34
Prior MI	10 (11%)	9 (15%)	1 (3%)	0.09	26 (14%)	18 (16%)	8 (12%)	0.51	0.45
Prior PCI	42 (45%)	28 (47%)	14 (41%)	0.67	79 (44%)	56 (49%)	23 (35%)	0.09	0.90
Prior CABG	1 (1%)	0 (0%)	1 (3%)	0.37	3 (2%)	2 (2%)	1 (2%)	> 0.99	> 0.99
PAD	10 (11%)	6 (10%)	4 (12%)	> 0.99	14 (8%)	11 (10%)	3 (5%)	0.26	0.50
FFR value	0.83 ± 0.02	0.83 ± 0.02	0.83 ± 0.01	0.68	0.90 ± 0.03	0.90 ± 0.03	0.90 ± 0.04	0.88	< 0.001
Target vessel									
LMCA and/or LAD	75 (81%)	49 (83%)	26 (76%)	< 0.001	103 (57%)	68 (60%)	35 (53%)	< 0.001	< 0.001
Circumflex	2 (2%)	0 (0%)	2 (6%)		20 (11%)	8 (7%)	12 (18%)		
RCA	15 (16%)	9 (15%)	6 (18%)		54 (30%)	36 (32%)	18 (27%)		
Others	2 (2%)	1 (2%)	1 (3%)		3 (2%)	2 (2%)	1 (2%)		
*Quantitative coronary angiography*									
Reference vessel diameter	2.6 ± 0.6	2.7 ± 0.6	2.6 ± 0.6	0.66	2.8 ± 0.7	2.8 ± 0.6	2.7 ± 0.8	0.58	0.25
Minimal lumen diameter	1.4 ± 0.5	1.4 ± 0.5	1.4 ± 0.4	0.85	1.5 ± 0.6	1.5 ± 0.6	1.5 ± 0.6	0.19	0.38
% Diameter stenosis	46.1 ± 13.9	46.6 ± 13.8	45.3 ± 14.1	0.65	46.6 ± 14.3	46.1 ± 14.5	47.4 ± 13.8	0.64	0.86
Lesion length	9.7 ± 5.2	9.9 ± 4.9	9.3 ± 5.7	0.35	9.7 ± 5.2	9.8 ± 5.5	9.5 ± 4.8	0.90	0.99
Laboratory data at FFR measurement									
Hemoglobin, g/dL	13.6 ± 1.6	13.7 ± 1.4	13.4 ± 2.1	0.64	13.9 ± 1.9	14 ± 1.8	13.7 ± 2.0	0.51	0.44
eGFR, mL/min/1.73m^2^	60.1 ± 22.7	58.5 ± 21.1	62.9 ± 25.1	0.58	60.8 ± 25.7	59.6 ± 25.9	62.9 ± 25.3	0.42	0.83
Total cholesterol, mg/dL	187.3 ± 54.9	177.1 ± 38.3	204.2 ± 71.8	0.02	181 ± 36.2	174.8 ± 37.2	191.9 ± 31.7	0.002	0.64
LDL-C, mg/dL	105.5 ± 38.5	100.3 ± 38.2	114.5 ± 37.4	0.06	98.5 ± 31	91.9 ± 31.9	110.0 ± 25.5	< 0.001	0.39
HDL-C, mg/dL	51.5 ± 18.6	53.1 ± 20.9	48.8 ± 13.6	0.57	56.6 ± 19	57.2 ± 19.5	55.6 ± 18.0	0.88	0.01
TG, mg/dL	167.9 ± 154.9	140.4 ± 71.7	214.8 ± 229.4	0.02	138.4 ± 100.7	135.6 ± 99	143.3 ± 103.4	0.89	0.003
HbA1c, %	6.2 ± 1.1	6.2 ± 0.9	6.3 ± 1.2	0.52	6.2 ± 1.2	6.2 ± 1.3	6.2 ± 1	0.57	0.36
*Laboratory data at 1 year after FFR measurement*									
Hemoglobin, g/dL	13.4 ± 1.7	13.4 ± 1.6	13.3 ± 1.9	0.93	13.5 ± 1.9	13.3 ± 1.8	13.8 ± 1.9	0.071	0.99
eGFR, mL/min/1.73m^2^	58.3 ± 21.9	57.6 ± 20.4	59.5 ± 24.3	0.83	56 ± 23.3	55.9 ± 23.5	56.1 ± 22.8	0.90	0.23
Total cholesterol, mg/dL	174.7 ± 44.6	153.9 ± 24.2	210.2 ± 48.9	< 0.001	175.5 ± 45.1	154.9 ± 27.9	209.2 ± 47.5	< 0.001	0.65
LDL-C, mg/dL	91.4 ± 28.4	74.4 ± 17.0	120.9 ± 18.4	< 0.001	94.9 ± 35.5	74.1 ± 16.7	130.8 ± 30.4	< 0.001	0.77
HDL-C, mg/dL	52.6 ± 15.2	53.3 ± 15.8	51.5 ± 13.9	0.74	56.1 ± 18	55.3 ± 17.6	57.4 ± 18.7	0.44	0.18
Triglyceride, mg/dL	174.5 ± 161.7	158.1 ± 135.4	202.9 ± 196	0.12	157 ± 131.7	156.9 ± 143.2	157.1 ± 108.4	0.52	0.50
HbA1c, %	6 ± 0.7	6.0 ± 0.7	6.0 ± 0.7	0.78	6.5 ± 6.6	6.8 ± 8.3	6.2 ± 1.2	0.85	0.54
*LDL-C value at MACCE event occurred*									
LDL-C, mg/dL	89.6 ± 26.1	77.0 ± 17.0	100.0 ± 27.8	0.02	87.3 ± 26.9	77.0 ± 19.1	107.0 ± 29.2	0.003	0.74
*Medications at FFR measurement*									
Aspirin	55 (59%)	39 (66%)	16 (47%)	0.08	83 (46%)	62 (54%)	21 (32%)	0.005	0.06
Clopidogrel	31 (33%)	21 (36%)	10 (29%)	0.65	49 (27%)	32 (28%)	17 (26%)	0.86	0.33
Prasugrel	10 (11%)	6 (10%)	4 (12%)	> 0.99	22 (12%)	18 (16%)	4 (6%)	0.06	0.84
Oral anticoagulant	7 (8%)	5 (8%)	2 (6%)	> 0.99	17 (9%)	11 (10%)	6 (9%)	> 0.99	0.66
Beta blocker	31 (33%)	22 (37%)	9 (26%)	0.36	62 (34%)	43 (38%)	19 (29%)	0.26	0.89
ACEi/ARB	32 (34%)	22 (37%)	10 (29%)	0.50	69 (38%)	47 (41%)	22 (33%)	0.34	0.60
MRA	6 (6%)	4 (7%)	2 (6%)	> 0.99	13 (7%)	9 (8%)	4 (6%)	0.77	> 0.99
Calcium channel blocker	31 (33%)	21 (36%)	10 (29%)	0.65	55 (31%)	35 (31%)	20 (30%)	> 0.99	0.68
Statins	50 (54%)	40 (68%)	10 (29%)	< 0.001	81 (45%)	62 (54%)	19 (29%)	< 0.001	0.20
Rosuvastatin	29 (31%)	24 (41%)	5 (15%)		45 (25%)	34 (30%)	11 (17%)		
Daily dose (mg)	3.7 ± 1.2	3.6 ± 1.2	4.0 ± 1.2	0.70	4.0 ± 1.8	3.8 ± 1.3	4.8 ± 2.7	0.77	> 0.99
Atorvastatin	7 (8%)	6 (10%)	1 (3%)		14 (8%)	10 (9%)	4 (6%)		
Daily dose (mg)	11.4 ± 3.5	11.7 ± 3.7	10 ± 0		9.3 ± 4.9	7.5 ± 2.5	13.8 ± 6.5		
Pitavastatin	13 (25%)	9 (15%)	4 (10%)		17 (10%)	15 (13%)	2 (3%)		
Daily dose (mg)	2.2 ± 0.5	2.0 ± 0	2.5 ± 0.9		2.3 ± 0.7	2.3 ± 0.7	2.0 ± 0		
Others	1 (1%)	1 (2%)	0 (0%)		5 (3%)	3 (3%)	2 (3%)		
Ezetimibe	7 (8%)	4 (7%)	3 (9%)		13 (7%)	9 (8%)	4 (6%)		
PCSK-9 inhibitor	0 (0%)	0 (0%)	0 (0%)	N.S	0 (0%)	0 (0%)	0 (0%)	N.S	N.S
EPA	8 (9%)	6 (10%)	2 (6%)	0.71	12 (7%)	10 (9%)	2 (3%)	0.22	0.63
Metformin	3 (3%)	1 (2%)	2 (6%)	0.55	13 (7%)	10 (9%)	3 (5%)	0.38	0.28
DPP4 inhibitor	10 (11%)	6 (10%)	4 (12%)	> 0.99	19 (11%)	13 (11%)	6 (9%)	0.8	> 0.99
SGLT2 inhibitor	3 (3%)	3 (5%)	0 (0%)	0.30	2 (1%)	2 (2%)	0 (0%)	0.53	0.34
PPI	33 (35%)	28 (47%)	5 (15%)	0.002	48 (27%)	36 (32%)	12 (18%)	0.056	0.66
Diuretic	11 (12%)	7 (12%)	4 (12%)	> 0.99	16 (9%)	12 (11%)	4 (6%)	0.42	0.52
*Medications at 1 year after FFR measurement*									
Aspirin	48 (52%)	34 (58%)	14 (41%)	0.14	75 (42%)	56 (49%)	19 (29%)		
Clopidogrel	28 (30%)	18 (31%)	10 (29%)	> 0.99	47 (26%)	37 (32%)	10 (15%)	0.01	0.48
Prasugrel	7 (8%)	5 (8%)	2 (6%)	> 0.99	17 (9%)	11 (10%)	6 (9%)	> 0.99	0.66
Oral anticoagulant	7 (8%)	4 (7%)	3 (9%)	0.70	20 (11%)	13 (11%)	7 (11%)	> 0.99	0.40
Beta blocker	33 (35%)	22 (37%)	11 (32%)	0.66	73 (41%)	57 (50%)	16 (24%)	< 0.001	0.44
ACEi/ARB	37 (40%)	28 (47%)	9 (26%)	0.052	73 (41%)	56 (49%)	17 (26%)	0.003	> 0.99
MRA	8 (9%)	6 (10%)	2 (6%)	0.71	14 (8%)	7 (6%)	7 (11%)	0.39	0.82
Calcium channel blocker	29 (31%)	20 (34%)	9 (26%)	0.5	48 (27%)	29 (25%)	19 (29%)	0.73	0.48
Statins	57 (61%)	45 (76%)	12 (35%)	< 0.001	112 (62%)	86 (75%)	26 (39%)	< 0.001	0.90
Rosuvastatin	32 (34%)	26 (44%)	6 (18%)		70 (39%)	52 (46%)	18 (27%)		
Daily dose (mg)	3.8 ± 1.2	3.9 ± 1.2	3.3 ± 1.2	0.53	4.0 ± 1.8	3.8 ± 1.2	4.6 ± 2.7	0.80	0.55
Atorvastatin	13 (14%)	10 (17%)	3 (9%)		16 (9%)	11 (10%)	5 (8%)		
Daily dose (mg)	10.4 ± 4.6	11.5 ± 4.5	6.7 ± 2.4		8.4 ± 3.8	8.2 ± 2.4	9.0 ± 5.8		
Pitavastatin	11 (12%)	8 (14%)	3 (9%)		23 (13%)	21 (18%)	2 (3%)		
Daily dose (mg)	2.5 ± 0.9	2.5 ± 0.9	2.7 ± 0.9		2.2 ± 0.9	2.3 ± 0.8	2.0 ± 0		
Others	1 (1%)	1 (%)	0 (0%)		3 (2%)	2 (2%)	1 (2%)		
Ezetimibe	12 (13%)	9(16%)	3(11%)		19 (11%)	13 (11%)	6 (9%)		
PCSK-9 inhibitor	1 (1%)	0 (0%)	1 (3%)	0.37	0 (0%)	0 (0%)	0 (0%)	NS	0.34
EPA	10 (11%)	8 (14%)	2 (6%)	0.32	17 (9%)	11 (10%)	6 (9%)	> 0.99	0.83
Metformin	11 (12%)	2 (4%)	1 (4%)	> 0.99	9 (5%)	7 (6%)	2 (3%)	0.49	0.76
DPP4 inhibitor	0 (0%)	6 (11%)	5 (18%)	0.52	15 (8%)	10 (9%)	5 (8%)	> 0.99	0.39
SGLT2 inhibitor	4 (4%)	3 (5%)	1 (3%)	> 0.99	6 (3%)	3 (3%)	3 (5%)	0.67	0.74
PPI	36 (39%)	25 (42%)	11 (32%)	0.38	67 (37%)	51 (45%)	16 (24%)	0.01	0.90
Diuretic	8 (9%)	4 (7%)	4 (12%)	0.46	17 (9%)	10 (9%)	7 (11%)	0.80	> 0.99

ACEi = angiotensin converting enzyme inhibitors; ARB = angiotensin II receptor blockers; BMI = body mass index; BNP = brain natriuretic peptide; BP = blood pressure; CABG = coronary artery bypass grafting; DPP4 = dipeptidyl peptidase-4; eGFR = estimated glomerular filtration rate; EPA = eicosapentaenoic acid; FFR = fractional flow reserve; HDL-C = high density lipoprotein cholesterol; HbA1c = hemoglobin A1c; LAD = left anterior descending coronary artery; LCx = left circumflex coronary artery; L/D = laboratory data; LDL-C = low-density lipoprotein cholesterol; LMCA = left main coronary artery; LVEF = left ventricular ejection fraction; MI = myocardial infarction; MLD = minimum lumen diameter; MRA = mineralocorticoid receptor antagonists; PCSK-9 = proprotein convertase subtilisin/kexin type 9; PAD = peripheral artery disease; PCI = percutaneous coronary intervention; %DS = % diameter stenosis; PPI = proton pump inhibitors; RCA = right coronary artery; SGLT2 = sodium glucose cotransporter 2.

#### Patients with an FFR of 0.81–0.85

Among the patients with an FFR of 0.81–0.85, the lower LDL-C group included significantly more male patients and was more likely to have patients with a lower body mass index and lower systolic blood pressure than the higher LDL-C group. The population that received statins for secondary prevention, that is, patients with a history of prior PCI and coronary artery bypass grafting, were comparably distributed in the lower and higher LDL-C groups. With respect to lipid profiles, there was no significant difference in LDL-C values at FFR measurement between the higher and lower LDL-C groups (*p* = 0.06). The mean LDL-C level declined from 100.3 to 74.4 mg/dL at 1 year after FFR measurement in the lower LDL-C group, while it rose from 114.5 to 120.9 mg/dL in the higher LDL-C group. Thus, the LDL-C value at 1 year after FFR measurement differed significantly between the higher and lower LDL-C groups (*p* < 0.001). The statin prescription rates both at FFR measurement and at 1 year after FFR measurement in the lower LDL-C group were significantly higher than those in the higher LDL-C group.

#### Patients with FFR > 0.85

On the other hand, patients with FFR > 0.85 showed no significant difference in clinical backgrounds between the higher and lower LDL-C groups, except for the distribution of the target vessel of FFR. FFR measurement was most often performed with the left ascending artery in both groups, and the percentage of measurements with this artery was higher in the lower LDL-C group. Consistent with the findings for patients with an FFR of 0.81–0.85, the mean LDL-C level declined from 91.9 to 74.1 mg/dL at 1 year after the FFR measurement in the lower LDL-C group, while it rose from 110.0 to 130.8 mg/dL in the higher LDL-C group. The statin prescription rates both at FFR measurement and at 1 year after FFR measurement were also significantly higher in the lower LDL-C group than in the higher LDL-C group.

### Prognoses

**Figure 2:**
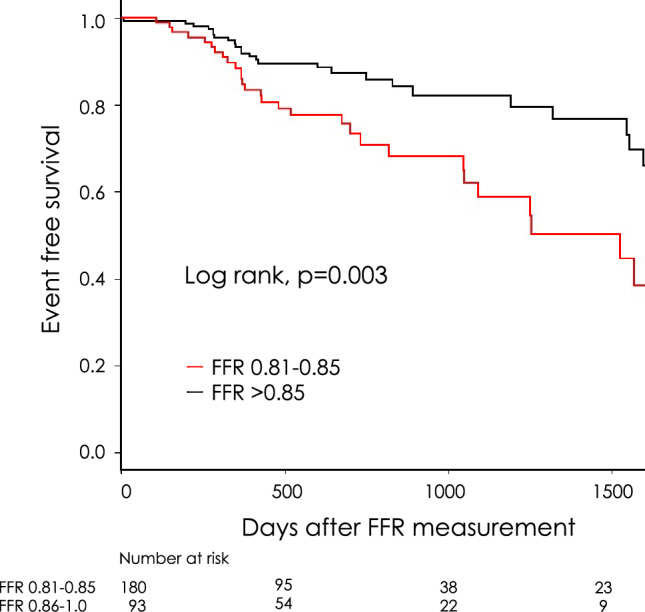
Major adverse cardiovascular and cerebrovascular events in patients with an FFR of 0.81–0.85 vs. those with FFR > 0.85. Kaplan–Meier event curves for major adverse cardiovascular and cerebrovascular events in patients with an FFR of 0.81–0.85 and FFR > 0.85. FFR = fractional flow reserve; LDL-C = low-density lipoprotein cholesterol.

During the median follow-up period of 663 days, the endpoints were observed in 30 patients (32%) with an FFR of 0.81–0.85 and 31 patients (17%) with FFR > 0.85. Kaplan–Meier analysis showed that the MACCE rate was significantly higher in patients with an FFR of 0.81–0.85 than in patients with FFR > 0.85 (log-rank, *p* = 0.003, Fig. 2). The hazard ratio (HR) for MACCE in patients with an FFR of 0.81–0.85 population was 2.14 (95% confidence interval [CI] 1.28–3.59) in comparison with the FFR > 0.85 population. The details of the endpoint are shown in Table 2. Unplanned revascularization for the target vessel which was measured FFR was the leading cause of MACCEs in each population. Non-fatal myocardial infarction during the observation period occurred in only one patient in each population. In the two patients who developed non-fatal myocardial infarction, the culprit lesion was located outside the target vessel that had been measured FFR in both cases. Cox regression analysis showed that the FFR value, T-C, LDL-C, and HbA1c levels were associated with MACCE (Table 3). The FFR value and HbA1c levels were independent predictors, even after adjusting for covariates.

**Table 2:** Details of major adverse cardiovascular and cerebrovascular events.

	FFR 0.81–0.85	FFR > 0.85
	n = 93	n = 180
Total events	30	31
Death	0	1
Non-fatal myocardial infarction	1	1
Ischemic stroke	5	2
Heart failure hospitalization	5	7
Unplanned revascularization of the vessel measured FFR	19	20

FFR = fractional flow reserve.

**Table 3:** Univariate Cox regression analysis in all patients, patients with FFR 0.81–0.85 and patients with FFR > 0.85.

Variables				All			FFR 0.81–0.85						FFR > 0.85					
	Univariate			Multivariable			Univariate			Multivariable			Univariate			Multivariable		
	OR	95% CI	*P* value	OR	95% CI	*P* value	OR	95% CI	*P* value	OR	95% CI	*P* value	OR	95% CI	*P* value	OR	95% CI	*P* value
Age, years	1.00	0.98–1.03	0.84				1.00	0.96–1.03	0.83				1.01	0.97–1.04	0.72			
Male	1.06	0.61–1.19	0.83				0.49	0.24–1.04	0.06	0.76	0.32–1.80	0.53	2.83	0.99–8.13	0.053	2.34	0.60–9.15	0.22
BMI	1.05	0.97–1.13	0.26				1.06	0.95–1.19	0.29				1.05	0.95–1.17	0.36			
Systolic BP	1.01	1.00–1.02	0.20				1.01	1.00–1.03	0.06	1.00	0.99–1.02	0.61	1.00	0.99–1.01	0.92			
Heart rate	1.01	0.99–1.02	0.54				1.01	0.99–1.03	0.30				1.00	0.97–1.03	0.88			
Smoking	0.65	0.35–1.19	0.16				0.55	0.22–1.36	0.20				0.78	0.34–1.79	0.56			
Atrial fibrillation	0.46	0.11–1.89	0.28				–	–	–				1.02	0.24–4.40	0.98			
Prior MI	0.74	0.35–1.57	0.43				1.13	0.39–3.29	0.82				0.54	0.18–1.60	0.26			
Prior PCI	0.82	0.49–1.38	0.46				0.71	0.34–1.50	0.37				0.95	0.46–1.97	0.90			
Prior CABG	0.33	0.04–2.53	0.28				3.04	0.41–22.81	0.28				–	–	–			
FFR value (per 0.1 increase)	0.39	0.20–0.76	0.006	0.48	0.57–2.73	0.11	0.87	0.07–11.65	0.92				0.41	0.11–1.46	0.17			
Target lesion in LAD	1.67	0.98–2.85	0.06	1.25	0.57–2.73	0.58	0.75	0.33–1.70	0.49				2.25	1.05–4.84	0.04	2.35	0.60–9.15	0.22
*Laboratory data and medication at 1 year after FFR measurement*																		
Hemoglobin	1.03	0.90–1.18	0.67				0.93	0.75–1.16	0.51				1.10	0.92–1.32	0.27			
eGFR	0.99	0.98–1.00	0.16				1.01	0.99–1.02	0.43				0.97	0.95–0.99	0.007	0.99	0.96–1.01	0.34
LDL-C	1.01	1.00–1.01	0.017	1.01	1.00–1.01	0.17	1.02	1.01–1.03	0.003	1.01	1.00–1.03	0.03	1.01	1.00–1.01	0.27			
HDL-C	0.99	0.97–1.01	0.34				0.99	0.96–1.01	0.32				1.00	0.97–1.02	0.85			
Triglyceride	1.00	1.00–1.002	0.29				1.00	1.00–1.003	0.049	1.00	0.99–1.00	0.38	1.00	0.99–1.00	0.29			
HbA1c	1.45	1.11–1.91	0.008	1.38	1.04–1.83	0.03	0.79	0.42–1.47	0.45				1.85	1.37–2.51	< 0.001	1.88	1.37–2.56	< 0.001
Statin	0.72	0.43–1.21	0.22				0.58	0.28–1.19	0.14				1.06	0.48–2.35	0.89			
Ezetimibe	0.98	0.90–1.07	0.62				1.00	0.88–1.13	0.98				0.97	0.86–1.09	0.60			

BMI = body mass index; BP = blood pressure; CI = confidence interval; PCI = percutaneous coronary intervention; CABG = coronary artery bypass grafting; eGFR = estimated glomerular filtration rate; FFR = fractional flow reserve; HbA1c = Hemoglobin A1c; HDL-C = high density lipoprotein cholesterol; LAD = left anterior descending coronary artery; LDL-C = low-density lipoprotein cholesterol; MI = myocardial infarction; OR = odds ratio.

**Figure 3:**
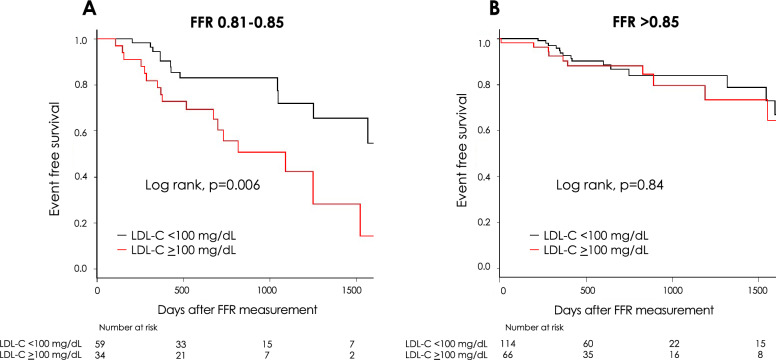
Major adverse cardiovascular and cerebrovascular events in the higher vs. lower LDL-C groups. Kaplan–Meier event curves for major adverse cardiovascular and cerebrovascular events in the higher and lower LDL-C groups with an FFR of 0.81–0.85 (A) and FFR > 0.85 (B). FFR = fractional flow reserve; LDL-C = low-density lipoprotein cholesterol.

In patients with an FFR of 0.81–0.85, the lower LDL-C group had a significantly lower MACCE rate than the higher LDL-C group (log-rank, *p* = 0.006, Fig. 3A). HR for MACCE in the lower LDL-C group was 0.36 (95% CI 0.17–0.77) compared to the higher LDL-C group. Univariate Cox regression analysis showed that LDL-C and TG levels were associated with MACCE in this population (Table 3). The prescription of statin was not a predictor for MACCE. Multivariable analysis revealed that the LDL-C level was an independent predictor, even after adjusting for covariates. On the other hand, there was no significant difference in the MACCE rate between the lower and higher LDL-C groups in patients with FFR > 0.85 (log-rank, *p* = 0.84, Fig. 3B). Among patients with FFR > 0.85, HR for MACCE in the lower LDL-C group was 0.94 (95% CI 0.43–2.06) compared to the higher LDL-C group. Univariate Cox regression analysis showed that LAD lesion, eGFR and HbA1c were predictors of MACCE in this population. HbA1c was an independent predictor for MACCE after multivariable analysis (Table 3). Finally, the *p*-value for the interaction between FFR populations (FFR of 0.81–0.85 or FFR > 0.85) and LDL-C groups (lower or higher LDL-C) was significant (*p* = 0.033).

## Discussion

### Principal findings of this study

Patients with FFR > 0.80 were successfully further stratified in relation to the risk of MACCE by using an FFR value of 0.85. The MACCE rate was significantly higher in patients with an FFR of 0.81–0.85 than in patients with FFR > 0.85. Uncontrolled LDL-C levels were associated with higher MACCE rates in patients whose PCI was deferred due to an FFR of 0.81–0.85. The leading cause of MACCE was unplanned revascularization. The novel aspect of this study lies in the revelation that lipid-lowering therapy, particularly in patients with deferred PCI lesions, is insufficient in real-world clinical settings. Moreover, we discovered that the intensity of LDL-C management significantly impacts the prognosis of patients with an FFR of 0.81–0.85. This group, deferred from PCI, represents a higher-risk population, further underscoring the vital role of diligent LDL-C management.

### High-risk population among patients who were deferred PCI based on FFR

The FAME trial concluded that FFR-guided PCI had an advantage over angiography-guided PCI in decreasing major adverse cardiac events for up to 2 years after the index procedure^13^. The DEFER trial reported that deferral of PCI for intermediate coronary stenosis based on an FFR ≥ 0.75 was associated with favorable outcomes during the very long-term follow-up period^14^. These trials established FFR measurement as a common tool for evaluating myocardial ischemia in a cath lab by a feasible and reproducible method with a clear cut-off point. Recently, the IRIS-FFR study analyzed the relationship between the FFR value and the HR for deferred versus revascularized lesions based on a multivariable model predicting major adverse cardiac events, including cardiac death, myocardial infarction, and repeat revascularization. The two lines intersected at an FFR value of 0.79^5^. An FFR of 0.75–0.80 is considered as a gray zone for the prognostic performance of PCI. Therefore, we adopted an FFR of 0.80 as the cut-off level for deferral of PCI, and patients with FFR > 0.80 were analyzed in this trial.

There is limited evidence to stratify the risk of MACCE development in patients with deferred PCI. To clarify the high-risk population among these patients, we performed further stratification of patients with FFR > 0.80. We adopted a cut-off value of 0.85 with regard for MACCE based on the results from the IRIS-FFR registry. The registry reported that the HR of major adverse cardiac events was 2.48 (95% CI 1.47–4.20) in lesions with an FFR of 0.81–0.85 and 1.60 (95% CI 0.91–2.80) in lesions with an FFR of 0.86–0.90, in comparison to lesions with an FFR of 0.91–1.0^5^. Our results showing that the MACCE rate was higher in patients with an FFR of 0.81–0.85 than in patients with an FFR > 0.85 were consistent with the results from the IRIS-FFR registry. Multivariable analysis also showed that FFR values could be predictor of MACCE in patients whose PCI was deferred on the basis of the FFR value. These results suggested that patients with an FFR of 0.81–0.85 were a high-risk population among patients whose PCI was deferred on the basis of FFR values, and that these patients should be followed up closely even after deferral of PCI.

### Lipid-lowering therapy in patients with deferred PCI on the basis of FFR

Secondary prevention of cardiovascular events by lipid control in patients who underwent PCI has been established based on evidence from various studies^6,10^; however, treatment guidelines for patients with deferred PCI based on FFR measurement have not been established. In this study, which evaluated the actual LDL-C management practices after FFR measurement in a real-world setting, we found that the mean LDL-C level at 1 year after FFR measurement was far from the recommended target level for secondary prevention. We have to recognize this current situation of clinical practice. Specifically, the mean LDL-C level increased in the higher LDL-C group at 1 year after FFR measurement. These findings suggest that in clinical practice, some patients without a prior history of cardiovascular disease may not receive adequate monitoring or sufficient lipid-lowering therapy. Of course, LDL-C level should be controlled under 70 mg/dL as a target level for secondary prevention in patients at high risk for cardiovascular events. However, in the real clinical settings, the numbers of patients with LDL-C level were very limited. Therefore, we adopted 100 mg/dL as a cut-off value of LDL-C in this study. In addition, patients did not receive sufficient lipid-lowering therapy regardless of the FFR value in the present study. There was no statistical difference in LDL-C values at 1 year after FFR measurement between patients with an FFR of 0.81–0.85 and those with FFR > 0.85.

In the current study, we reported the different prognostic effects of LDL-C management according to the patients’ FFR values. An uncontrolled LDL-C level was associated with a higher MACCE rate in patients with an FFR of 0.81–0.85, who were at a relatively higher risk of MACCE among patients whose PCI was deferred. In contrast, the LDL-C level was not associated with the MACCE rate in patients with FFR > 0.85, who were at a relatively lower risk of MACCE among patients whose PCI was deferred. Multivariable Cox regression analysis showed that the LDL-C level was an independent predictor even after adjusting for covariates in patients with an FFR of 0.81–0.85, but not in patients with FFR > 0.85. Additionally, the interaction between the FFR population (FFR of 0.81–0.85 or FFR > 0.85) and LDL-C groups (lower or higher LDL-C groups) was statistically significant. These results indicate the importance of LDL-C control, especially in patients with an FFR of 0.81–0.85. Of note, these results do not indicate that LDL-C management is less important in patients with FFR > 0.85. All patients with intermediate coronary stenosis, which were performed FFR measurement, should be received intensive LDL-C management. In a previous study by Nakamura et al.^15^, uncontrolled LDL-C levels were found to be associated with a higher risk of deferred lesion failure and any unplanned revascularization in patients with deferred lesion. Despite the well-established importance of LDL-C management in patients with CAD, our study findings suggest that there may be insufficient LDL-C management in clinical practice after FFR measurement. Therefore, as the first step, the risk stratification presented in this study is an important new perspective.

A close correlation has been reported between achieved LDL-C levels and the changes in the atheroma volume in a prior trial using intravascular ultrasound^16^. According to the GLAGOV randomized trial, an intensive LDL-C-lowering therapy with PCSK9i resulted in a greater decrease in the percent atheroma volume than a normal control^17^. One report described LDL-C control and FFR values for lesions after PCI, and showed that an LDL-C value over 70 mg/dL was associated with an increased FFR value for the target vessel at 18 months after PCI^18^. Since insufficient LDL-C control was associated with a high MACCE rate in patients with an FFR of 0.81–0.85 but not in those with FFR > 0.85, insufficient LDL-C could cause an atheroma volume increase or a destabilization of lipid plaque, leading to myocardial ischemia. These results suggest that patients whose PCI was deferred on the basis of an FFR of 0.81–0.85 should be followed up closely and should receive sufficient lipid-lowering therapy.

### Study limitations

This study has several limitations that should be acknowledged. First, this was a retrospective observational study performed in a single center with a relatively small number of patients. The reason that LDL-C emerged as an independent predictor for MACCE, while statin prescription did not, might be attributed to the limited sample size of the study. In the entire study population, the hazard ratio for statin prescription was 0.72 (95% CI 0.43–1.21), which did not reach statistical significance. It is conceivable that with a larger cohort, the risk reduction associated with statin prescription could achieve statistical significance**.** Second, the cut-off LDL-C value was 100 mg/dL in our study, although the guidelines recommended LDL-C < 70 mg/dL as the target for patients with a very high cardiovascular event risk, such as patients with previous acute coronary syndrome and familial hypercholesterolemia^10^. The reason was mentioned in the discussion part. However, the average LDL-C value in the lower LDL-C group was approximately 74 mg/dL in both FFR groups. Thus, LDL-C control in the lower LDL-C group nearly achieved the strict target under the guideline recommendations. Third, our study excluded patients with multiple intermediate stenoses that required separate FFR measurements because they could not be allocated into subgroup based on FFR value. Therefore, patients with a relatively lower severity of atherosclerosis may have been selected for analysis in the current study. Fourth, we need to acknowledge a limitation concerning our patient classification into lower and higher LDL-C groups based on LDL-C values obtained 1 year post-FFR measurement. We must note that nine patients in the FFR 0.81–0.85 group and ten patients in the FFR > 0.85 group encountered MACCE within the first year after FFR measurement. Despite this, the trend of LDL-C values at the time of MACCE was found to align with the pattern observed at the one-year mark post-FFR measurement. Considering the lower-the-better strategy for LDL-C management, a randomized controlled study to verify the impact of strict LDL-C management in high-risk patients with deferred PCI is warranted.

## Conclusions

Among patients with deferred PCI after FFR measurement, MACCEs were more frequent in patients with FFR 0.81–0.85 than in those with FFR > 0.85. Uncontrolled LDL-C levels were associated with higher event rates in patients with deferred PCI due to FFR 0.81–0.85 but not in those with FFR > 0.85. The insights gained from our study advocate for rigorous LDL-C management, involving comprehensive lipid-lowering therapy and meticulous monitoring, especially in patients with coronary artery stenosis exhibiting lower FFR values, even after deferral from PCI. This is integral to optimize patient outcomes and ensure the best possible care in this specific patient cohort.

## Data Availability

The datasets used and/or analyzed during the current study available from the corresponding author on reasonable request.

## Abbreviations

ACS: Acute coronary syndrome
BNP: Brain natriuretic peptide
CAD: Coronary artery disease
eGFR: Estimated glomerular filtration rate
FFR: Fractional flow reserve
HDL-C: High density lipoprotein cholesterol
LDL-C: Low-density lipoprotein cholesterol
MACCE: Major adverse cardiovascular and cerebrovascular events
PCI: Percutaneous coronary intervention
T-C: Total cholesterol
TG: Triglyceride
